# Autistic adult outcomes on weight and body mass index: a large-scale online study

**DOI:** 10.1007/s40519-019-00695-8

**Published:** 2019-05-07

**Authors:** Felicity Sedgewick, Jenni Leppanen, Kate Tchanturia

**Affiliations:** 1 King’s College London, Institute of Psychiatry, Psychology and Neuroscience, Psychological Medicine, London, UK; 2 South London and Maudsley NHS Trust EDU, London, UK; 3 Psychology Department, Illia State University, Tbilisi, Georgia

**Keywords:** Autism, Adult outcomes, Obesity, Eating, Weight, Mental health

## Abstract

**Purpose:**

There has been a wealth of work on the weight outcomes of autistic children and young people, generally finding that they are more likely to be overweight or obese than their non-autistic counterparts. There has not been the same focussed study of the weight outcomes of autistic adults, however. This study, therefore, sought to examine the relationship between weight outcome and being autistic in adults.

**Methods:**

Data were collected as part of an online study looking at eating, autism, and relationships. 665 people gave demographic and mental health information, and group differences and robust regressions were conducted.

**Results:**

Autistic adults were more likely to be in non-healthy weight categories than their non-autistic counterparts, i.e., more likely to be underweight, overweight, or obese. There were no interactions between autism status and mental health impacting BMI, although both anxiety and depression predicted higher BMI in the sample overall.

**Conclusions:**

We conclude that while some weight patterns from childhood and adolescence continue into adulthood for autistic individuals, this is not necessarily a straightforward picture, and would benefit from further in-depth and qualitative study to understand the processes at play. The lack of interactions between mental health and autism, however, should provide professionals with confidence in supporting healthy weight management among autistic people.

**Level of evidence:**

Level III, cohort study.

## Introduction

When discussing the weight outcomes of autistic people, there is a wealth of work showing that children and young people on the spectrum are more likely to be overweight or obese than their non-autistic peers [1, 2]. There are a wide range of reasons for being overweight, but some of the most common factors—consuming more calories than you burn, low levels of physical activity—may particularly affect autistic people [3]. What has not previously been thoroughly explored, however, is whether these patterns extend into adulthood, or their relationships with factors known to influence weight outcomes in non-autistic people.

Autistic children and young people often have very different diets to non-autistic children and young people, consuming a more limited range of foods [4], although this pattern is not universal [5]. Those foods which are often preferred by autistic children and young people tend to have a higher calorie count or have lower nutritional value [6]. Although it is common for children and young people to enjoy hobbies such as videogames, autistic children and young people engage in less physical activity than their non-autistic peers [7], tending to spend their time with their autistic and non-autistic friends in more sedentary activities [8].

To our knowledge, there are to date no studies which frame their work specifically as studying weight outcomes in autistic adults, but there is work which has included these data in exploring health outcomes generally. This work finds that autistic adults are more likely to be overweight/obese than non-autistic adults, along with also being more likely to have a wide range of health complaints [9, 10]. Most adult outcome work has focussed on cognitive and social outcomes [11–13], but health outcome work generally shows that autistic adults have more difficulties. Work in non-autistic populations has shown that people with multiple health challenges are more likely to be in the overweight/obese categories [14, 15], and this is likely to also be the case for autistic adults.

Alongside physical health, mental health conditions have also been shown to be linked to higher BMI (body mass index) [16, 17] in non-autistic individuals. Autistic people are more likely to experience poor mental health than their non-autistic counterparts, with higher levels of anxiety [18, 19] and depression [20, 21] being particularly common. It is, therefore, possible that this interaction between mental health issues and increased BMI is also the case for autistic adults, but this has not been previously investigated.

One mental health condition which is by definition associated with low rather than high BMI is anorexia nervosa (AN), and connections between autism and AN have been suggested and explored since the 1980s [22]. This exploration has normally taken the form of conceptualising AN as a ‘female form’ of autism [23], with similarities in cognitive profiles—set-shifting difficulties [24], detail-focussed processing [25, 26], and theory of mind challenges [27]—and in social difficulties [28, 29]. These studies have, however, exclusively used female participants, as women are more likely to develop AN [30], compared to autism being more commonly diagnosed in males due to well-recognised diagnostic biases [31]. Furthermore, research looking at the overlap between the two conditions has very much focussed on the presence and level of autistic traits in AN populations, with some studies finding up to 23% of women receiving in-patient treatment for AN meet clinical criteria for autism [32, 33]. This means that to date, there is no published research on how likely autistic people are to meet the criteria for AN of having a BMI below 18.5 [34].

This study, therefore, sought to examine all weight category outcomes of autistic and non-autistic adults, and the potential impact of mental health on these outcomes. Our hypotheses were:

1. Autistic people would on average have a higher BMI than non-autistic people, and would be more likely to be in the higher weight categories,
2. People with more mental health difficulties would have a higher BMI than those without mental health difficulties,
3. There would be an interaction between autism status and mental health resulting in higher BMI outcomes

## Method

### Participants

Six hundred and sixty-five people between the ages of 18 and 81 were included in the analysis, after the exclusion of 276 participants who self-reported having an eating disorder diagnosis. Of these 665, 335 (50.38%) reported that they were autistic, and 330 (49.62%) reported no autism diagnosis. Gender, ethnicity, employment status, and other reported diagnoses among the two groups can be seen in Table 1.

**Table 1:** Demographic characteristics of the sample by group (autistic, non-autistic)

	Autistic (*n* = 335)	Non-autistic (*n* = 330)
Age		
Range	18.12–71.42	18.29–81.29
M (SD)	34.06 (10.86)	32.67 (11.25)
Gender		
Male	53 (15.82%)	53 (16.06%)
Female	195 (58.21%)	266 (80.61%)
Non-binary	86 (25.67%)	12 (3.63%)
Ethnicity		
White	246 (73.43%)	262 (79.39%)
Asian	8 (2.38%)	24 (7.27%)
Black	3 (0.90%)	3 (0.91%)
Latinx	3 (0.90%)	1 (0.30%)
Mixed	14 (4.18%)	9 (2.73%)
No answer	59 (17.61%)	31 (9.39%)
Employment status		
Full time	89 (26.57%)	177 (53.63%)
Part time	40 (11.94%)	27 (8.18%)
Student	60 (17.91%)	91 (27.57%)
Self-employed	39 (11.64%)	14 (4.24%)
Unemployed	73 (21.79%)	12 (3.63%)
Retired	8 (2.38%)	4 (1.21%)
Other	23 (6.87%)	5 (1.52%)
Eating-related diagnoses		
Diabetes	21 (6.27%)	4 (1.21%)
Coeliac disease	3 (0.89%)	1 (0.30%)
Crohn’s disease	2 (0.60%)	1 (0.30%)
Ehlers–Danlos syndrome	2 (0.60%)	0 (0.00%)
Food intolerances/allergies	104 (31.04%)	48 (14.54%)
Irritable bowel syndrome	16 (4.77%)	8 (2.42%)
AQ score		
Range	4–28	0–25
M (SD)	20.98 (3.83)	8.93 (5.68)

Participants were recruited online through social media (Twitter, Facebook) and through online advertising on the King’s College website and email circulars. Ethical approval was obtained from the King’s Psychiatry, Nursing and Midwifery Research Ethics Committee (LRS-17/18-5292). All participants read a full information page before taking part in the study, and were informed that completing the study would be taken as consent for the use of their data. Participants also completed a written informed consent page before starting the survey. All procedures were conducted in accordance with the latest version of the Declaration of Helsinki.

### Measures

#### Demographics

Participants completed a demographics questionnaire, including their age, height, weight, ethnicity, and employment status.

*AQ:* The Autism Quotient-28 item version [35] is a 28-item self-report screening questionnaire assessing the presence and level of autism symptomatology an individual experiences. Answers are given on a Likert scale from ‘Very accurate’ to ‘Very inaccurate’ and are then scored 1 or 0 depending on the direction of the question. Higher scores reflect more autistic symptomatology.

#### EDE-Q

The Eating Disorder Examination Self-report Questionnaire [36] is a 36-item self-report questionnaire assessing eating disorder psychopathology over the past 28 days. Participants score the frequency of their behaviours or thoughts from ‘0’ days’ to ‘Every day’. Higher scores reflect greater eating disorder symptomatology.

#### HADS

The Hospital Anxiety and Depression Scale [37] is a 14-item self-report questionnaire assessing levels of both anxiety and depression over the past 2 weeks. Answers are scored from 0 (not anxious/depressed) to 3 (very anxious/depressed) on each item for a maximum score of 42. Higher scores reflect higher anxiety and depression levels.

### General procedure

Participants all completed the study online, at their own pace and in a place of their preference. The data were collected as part of a larger study. Participants completed demographic information, the AQ, the EDE-Q, and the HADS online.

### Data analysis

All data analyses were conducted with R [38]. Group differences in demographic and clinical characteristics were explored with *t* tests. Weight category outcomes according to autism status were investigated using ordinal logistic regression [39]. Impact of self-reported mental health and autism status on BMI outcomes was investigated using robust regression [40]. Separate robust regression models were built to examine the impact of self-reported anxiety (HADS anxiety), depression (HADS depression), and eating disorder symptomatology (EDEQ total) along with autism status on BMI. Separate Spearman’s correlation analyses were conducted to examine associations between AQ-28 scores and BMI within the two groups. Significance level was set at *p* < 0.05.

## Results

### Demographics

Participants were matched on age, *t*(664) = − 1.62, *p* = 0.11. They were not matched on AQ score, with those who reported being autistic scoring significantly higher than those who reported being non-autistic, *t*(664) = − 31.22, *p* < 0.001.

### Weight outcomes

The numbers of autistic and non-autistic people in each weight category can be seen in Table 2. Autistic people had a higher average BMI than non-autistic people, *t*(664) = − 4.03, *p *< 0.001. Within the autistic group, higher AQ scores were correlated with lower BMI, *r* = − 2.51, *p* = 0.01. Within the non-autistic group, there was no correlation between AQ score and BMI, *r* = 1.43, *p* = 0.15.

**Table 2:** BMI and weight category outcomes by group (autistic, non-autistic)

	Autistic (*n* = 335)	Non-autistic (*n* = 330)
BMI		
Range	14.56–72.31	15.06–63.82
M (SD)	28.01 (8.36)***	25.66 (6.49)
Weight category		
Underweight	18 (5.37%)***	9 (2.73%)
Healthy weight	126 (37.61%)	189 (57.28%)
Overweight	86 (25.67%)***	71 (21.52%)
Obese	104 (31.04%)***	61 (18.48%)

*Denotes significance at the 0.05 level

**Denotes significance at the 0.01 level

***Denotes significance at the 0.001 level

A categorical outcome regression showed that being autistic had a significant impact on likely weight category outcome. Taking healthy weight as the baseline, autistic people were more likely to be either underweight (*t* = − 14.20, *p* < 0.001), overweight (*t *= 3.26, *p* < 0.001), or in obesity range (*t *= 11.72, *p *< 0.001) than non-autistic people. Being autistic was linked to a 58.84% greater chance of being in the overweight/obese weight categories than healthy weight category.

### Mental health and BMI

Scores on mental health and clinical measures can be seen in Table 3. Autistic people scored more highly than non-autistic people on both the anxiety, *t*(664) = − 12.35, *p* < 0.001, and depression subscales of the HADS, *t*(664) = − 10.85, *p* < 0.001. There were no significant differences between the groups on the EDE-Q, *t*(664) = − 1.14, *p* = 0.25.

**Table 3:** Mental health and clinical scores by group (autistic, non-autistic)

	Autistic (*n* = 335)	Non-autistic (*n* = 330)
HADS anxiety		
Range	0–21	0–21
M (SD)	11.81 (4.45)***	7.55 (4.44)
HADS depression		
Range	0–21	0–18
M (SD)	7.23 (3.87)***	4.13 (3.50)
EDEQ		
Range	0–5.68	0–5.68
M (SD)	1.77 (1.39)	1.66 (1.21)

*Denotes significance at the 0.05 level

**Denotes significance at the 0.01 level

***Denotes significance at the 0.001 level

There was a significant main effect of autism status controlling for anxiety on BMI, *F*(1) = 9.70, *p* = 0.002, but no main effect of anxiety score, *F*(1) = 1.77, *p* = 0.18, and no interaction between autism status and anxiety score, *F*(1) = 0.83, *p* = 0.36.

There was significant main effect of autism status on BMI controlling for depression, *F*(1) = 5.800, *p* = 0.2, and also a significant main effect of depression score on BMI, *F*(1) = 13.17, *p* < 0.001, but no significant interaction between autism status and depression score, *F*(1) = 0.02, *p* = 0.90.

There was a significant main effect of autism status controlling for EDE-Q, *F*(1) = 16.15, *p* < 0.001, and also a main effect of EDE-Q score on BMI, *F*(1) = 129.46, *p* < 0.001, but no significant interaction between autism status and EDE-Q score, *F*(1) = 0.65, *p* = 0.42.

## Discussion

The findings of this study suggest that autistic adults are less likely than non-autistic adults to have a healthy BMI, being more likely to be both under- and overweight. This finding draws together the results of two disparate fields of research—that on obesity in autism, and that on links between anorexia and autism—in ways which will be discussed in detail below. Higher BMI was linked to mental health difficulties in both autistic and non-autistic participants, but interestingly, there was no combined effect of being autistic and having mental health issues on BMI.

Our finding that autistic adults were found to be more likely to have BMI scores in the overweight or obese categories than non-autistic people is in line with earlier work which has shown that autistic children, adolescents, and adults are more likely to be overweight or obese than their non-autistic counterparts [2, 3, 10]. This is the first large-scale online study to look at adult weight outcomes in autistic people, and the results suggest that the weight patterns established in early life might continue into adulthood. In young autistic people, being overweight/obese is linked to lower activity levels [7], greater caloric intake [5], and preferences for low-nutrition foods [4]. Considering that difficulties with and a dislike of change is a well-recognised feature of autism [34], it may well be that these behavioural patterns are continued into adulthood, meaning that overweight/obese status is also maintained.

The seemingly contrary finding that autistic people are also more likely to be in the underweight category than the healthy weight category, however, can also be explained in terms of behaviour patterns being maintained from adolescence. There is a wealth of work examining links between anorexia nervosa, an illness characterised by low body weight and the pursuit of extreme thinness [34], and autism or autistic traits [32, 33, 41]. This research has shown that up to 23% of women with severe and enduring anorexia, i.e., those who maintain very low BMI for many years, have clinically significant levels of autism traits [32]. This association between autism and anorexia has been linked to similar cognitive profiles in the two conditions [27, 42–44]. It may, therefore, be that those autistic people, especially autistic women, who develop an eating disorder are more likely to fall into the lowest BMI category anyway and, therefore, find it hardest to change their behaviour pattern to achieve a healthy weight regardless of ongoing ED. There is also some evidence that children with developmental disabilities are more likely to be in the under- as well as overweight categories [45], a pattern which may also be maintained into adulthood.

This interpretation is supported by the finding that within the autistic group, higher AQ score was correlated with lower BMI, suggesting that more severely autistic people are more likely to have restricted calorie intake. Wider literature proposes a range of possible reasons for this, particularly around sensory sensitivities. That autistic people are often hyper- or hypo-sensitive to touch, taste, smell, and light is well-documented and was included as a diagnostic criterion in the new DSM-V [34]. These sensitivities may contribute to the restricted dietary range often seen in autistic children [4], and potentially result in the same in autistic adults, as people seek to avoid unpleasant or overstimulating foods. This in turn potentially leads to more acute malnutrition and low BMI amongst those with more sensory challenges associated with higher levels of autism symptomatology.

These sensory sensitivities may also play a role in the higher numbers of autistic adults who were in the overweight/obese categories as well as those in the underweight category. Just as some autistic people may be underweight due to sensory avoidance of unpleasant or overwhelming stimuli, some autistic people may be overweight/obese due to sensory seeking of foods which are pleasant to them. Considering work which has shown that, regardless of autism status, people find more emotional satisfaction from foods associated with their childhoods [46, 47] and that autistic children often have diets containing relatively high calorie foods [6], it is reasonable to assume that autistic adults who sensory seek through food tend to go for those same high calorie items, contributing to weight gain.

It was somewhat surprising that BMI outcome was not impacted by the interaction between autism and mental health. Individuals with mental health issues are often found to have higher BMI than those without mental health issues [16, 17], as are autistic people [10]. Considering that autistic people are more likely to have mental health issues than non-autistic people [18], a pattern which was also present in this study, it would be reasonable to predict that autistic people with mental health issues would have higher BMI scores than non-autistic people with mental health issues or autistic people without mental health issues. That this is not the case, however, is promising in that it suggests that there are not a set of unique processes occurring for autistic people with mental health issues regarding their weight outcomes, and therefore, weight interventions which already exist can be implemented with confidence. It is also the case that anxiety levels in our non-autistic sample were higher than the expected population norm, sitting at 7.55 in our sample compared to 4.4–5.0 in normative data [37]. This may be because the majority of participants were female, and women are known to experience higher levels of anxiety than men [48].

While this is generally a strong study due to the large sample size, there are some limitations to the research. First, the data come entirely from self-report, which raises the possibility of inaccurate answers, especially regarding weight. It is common for people to be unclear as to their weight in a way that it is not for their height, and therefore, it may be that there are some people who either under- or over-reported their weight. Despite this, the broad range of BMI scores in both the autistic and non-autistic samples suggest that the overall sample is representative even if individual scores are somewhat off. Second, the groups were not matched on demographic variables such as gender or employment status. This is to be expected, however, considering work showing that autistic people are more likely to be gender non-conforming than non-autistic people [49–51] and that they can struggle to maintain full-time employment [52, 53], and therefore, these differences are representative of the population. Third, this study lacks any qualitative data, so we are unable to discuss what experiences and drivers people felt contributed to their weight in their own words. Future work exploring this topic would be valuable, as people cannot be supported in living healthier lives if we do not know what causes unhealthy behaviours in the first place. Fourth, this work captures people’s weights at just one point in time, rather than being longitudinal, and does not track the many factors which are known to promote weight gain over time. Finally, there are potential selection biases in online recruitment, such as the reliance on the literacy and written communication abilities of participants which may mean that our findings are not representative of those autistic people who have difficulties with these media. It is also possible that our participants were individuals who have an interest in the topics of autism and weight, although the wide range of BMI scores and the preponderance of those in the Normal weight category suggest that this is not necessarily a key bias present in the sample.

In conclusion, our data are the first large-scale study which shows that autistic adults are more likely to be in all weight categories considered ‘unhealthy’, i.e., underweight, overweight, and obese, than they are to be in the healthy weight category. This is in line with a wealth of work which has examined the weight outcomes of autistic children and adolescents and extends our understanding of health across the lifespan for autistic people. In this study, there were no impacts on BMI from the interactions between self-reported mental health and autism status, suggesting that autistic people would likely benefit from the same interventions as non-autistic people without the assumption that they will inherently have mental health challenges. Future work should include qualitative explorations of the factors and experiences which autistic adults themselves feel contribute to their weight status to understand how to best support them regarding healthier choices, along with understanding whether autistic people want this kind of support at all.
